# Accuracy of cited “facts” in medical research articles: A review of study methodology and recalculation of quotation error rate

**DOI:** 10.1371/journal.pone.0184727

**Published:** 2017-09-14

**Authors:** Scott A. Mogull

**Affiliations:** Texas State University, San Marcos, Texas, United States of America; University of Illinois-Chicago, UNITED STATES

## Abstract

Previous reviews estimated that approximately 20 to 25% of assertions cited from original research articles, or “facts,” are inaccurately quoted in the medical literature. These reviews noted that the original studies were dissimilar and only began to compare the methods of the original studies. The aim of this review is to examine the methods of the original studies and provide a more specific rate of incorrectly cited assertions, or quotation errors, in original research articles published in medical journals. Additionally, the estimate of quotation errors calculated here is based on the ratio of quotation errors to quotations examined (a percent) rather than the more prevalent and weighted metric of quotation errors to the references selected. Overall, this resulted in a lower estimate of the quotation error rate in original medical research articles. A total of 15 studies met the criteria for inclusion in the primary quantitative analysis. Quotation errors were divided into two categories: content ("factual") or source (improper indirect citation) errors. Content errors were further subdivided into major and minor errors depending on the degree that the assertion differed from the original source. The rate of quotation errors recalculated here is 14.5% (10.5% to 18.6% at a 95% confidence interval). These content errors are predominantly, 64.8% (56.1% to 73.5% at a 95% confidence interval), major errors or cited assertions in which the referenced source either fails to substantiate, is unrelated to, or contradicts the assertion. Minor errors, which are an oversimplification, overgeneralization, or trivial inaccuracies, are 35.2% (26.5% to 43.9% at a 95% confidence interval). Additionally, improper secondary (or indirect) citations, which are distinguished from calculations of quotation accuracy, occur at a rate of 10.4% (3.4% to 17.5% at a 95% confidence interval).

## Introduction

The pages of any book, tract or article dealing with medicine are apt to be profusely sprinkled with numerical superscripts (or their equivalents) guiding the reader to a reference list. Not only does the liberal presence of such reference numbers impart an aura of scholarship, but their judicious placement after this or that assertion subtly suggests documented validity. But watch out—those little numbers may be no more than the trappings of credibility. The primary sources cited may be misquoted, inapplicable, unreliable and occasionally even imaginary.[1]

FJ Ingelfinger

Editor (emeritus)

The New England Journal of Medicine

Since Ingelfinger’s initial observations and commentary on the accuracy and reliability of quoted assertions in the medical literature, researchers have been systematically investigating the prevalence of inaccurately cited “facts” in published journal articles. Such quotation errors undermine the scientific argument and foundation for the research being reported in the article and, moreover, may distort[2] and amplify[2–4] false information throughout the medical literature. While quotation errors are concerning for all readers, these errors are a particular problem for physicians and the general public who are not focused on the scientific study of a narrow research topic and thus are less prone to identify rhetorically misleading statements or outright factual errors.[5]

In the first, and only, systematic investigation of the overall rate of quotation errors representing the general medical literature,[6] the authors estimated that 15% of all cited assertions in published journal articles (including original research articles and literature reviews) were inaccurate. Subsequent studies on quotation errors were more focused—generally examining quotation errors in original research articles within particular medical specialties—although some studies were highly focused case studies on the quotation error rate within a specific journal or tracking the error rate and variations of a specific “fact” throughout the literature.

Authors of previous review articles have estimated the overall rate of quotation errors in the medical literature by combining the rates reported from all studies providing a quantitative estimate of quotation error rate in the medical literature (which included applied health fields). In a review of studies published through 2006–7, the quotation error rate median was estimated at 20%.[7] In a subsequent review of studies published through 2011, a similar quotation error rate mean was estimated at 19.7%.[8] In the latest review of studies published through 2014, and notably the most robust analysis, the total quotation error rate provided in the primary analysis was estimated at 25.4%.[5] As noted by the authors of previous reviews,[5, 7–9] the original studies had different criteria for quotation errors and different methods to calculate the total quotation errors rate. In the most recent review,[5] the authors adjusted for these inconsistencies through a statistical measure of bias, which was based whether the original studies randomly selected references (quotations) to evaluate and the number of independent raters of each quotation error.

However, the predominantly quantitative approaches used in previous reviews were limited because the measures employed did not account for the full range of inconsistencies in the methods and calculations in the original studies. Thus, the goal of this study is to determine the overall rate of quotation errors in medical journal articles after accounting for the methodological differences in the original studies. Unlike other reviews, this review is predominantly a qualitative analysis of methodological differences in the original studies. In this review, the original studies are sorted by research design, the raw data is regrouped according to consistent definitions and criteria of quotation errors, and then the overall quotation error rate is recalculated. The primary outcome of this investigation is a more precise, yet narrower in scope, estimate of the rate of quotation errors in original research articles published in medical journals. A secondary outcome is an analysis of the methods used in original studies of quotation errors, which may be useful for standardizing future investigations and enabling, to some degree, more reliable comparison between quotation error rates in different medical specialties.

## Methods

### Search strategy

For this analysis of quotation errors, I conducted a systematic keyword search of the PubMed/MEDLINE database followed by manual searching of reference lists. The identification and screening phase was originally conducted on December 29, 2015, when I searched PubMed/MEDLINE using 11 keywords/algorithms with an unrestricted date range or article type. The 11 keywords/algorithms were: quotation AND accuracy, “quotation accuracy,” “reference accuracy,” “citation accuracy,” “quotation error,” quotation AND error, (reference AND error) AND “bibliometrics”[MeSH Terms], “reference error,” “citation error,” (citation analysis) AND bibliography[MeSH Terms], and (reference AND accuracy) AND “bibliometrics”[MeSH Terms].

**Fig 1:**
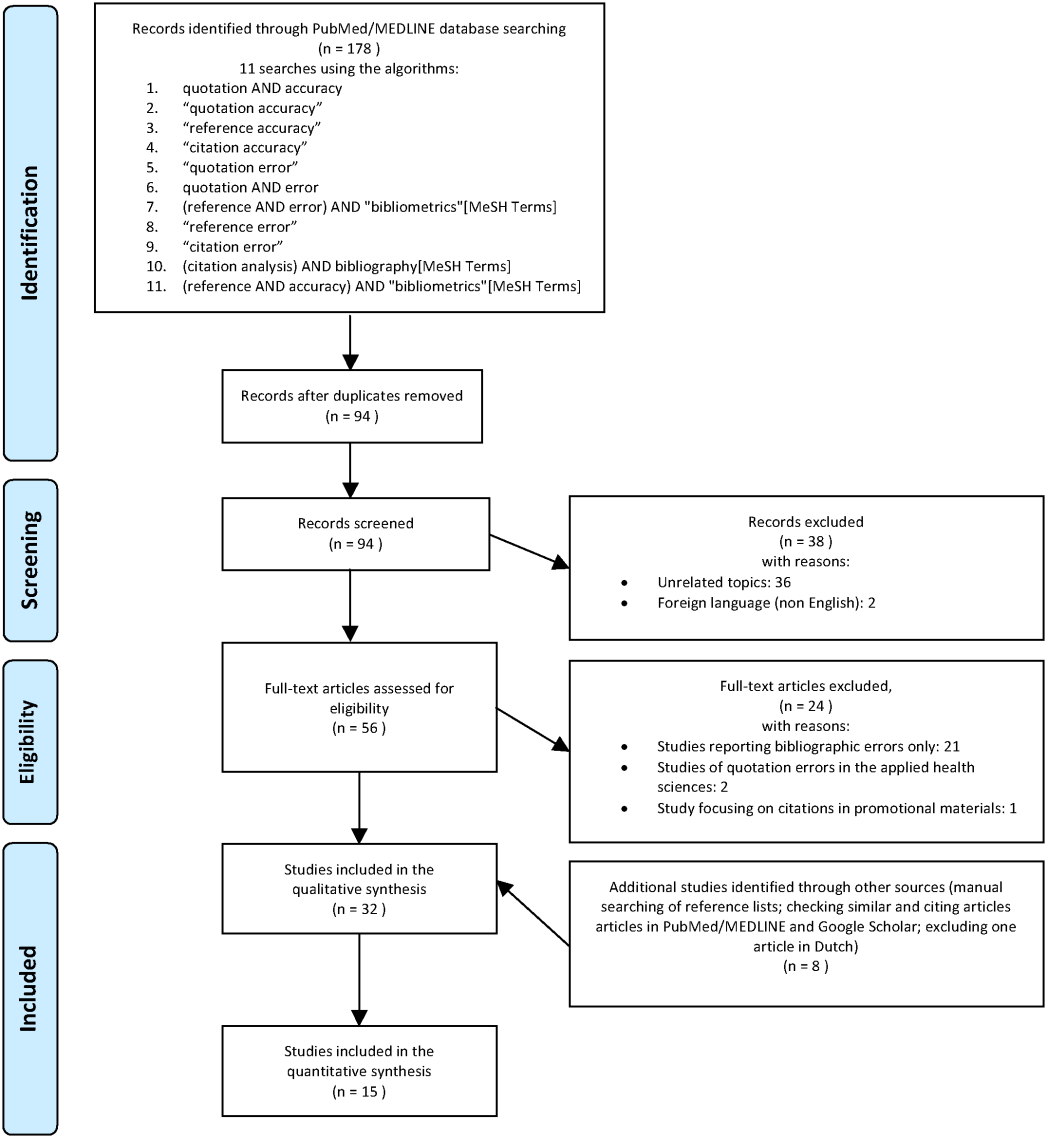
Identification and selection of articles on quotation errors in the medical literature.

The initial searches yielded 178 results that were merged into a single PubMed collection, which excluded 84 duplicate records (Fig 1). The 94 remaining records were manually screened and 63 records were excluded from further analysis. Most of the articles were excluded because the topics were unrelated to this investigation (n = 62). Two PubMed/MEDLINE records were for the same article, so an additional record was excluded (n = 1). The complete text of the remaining 31 articles were analyzed. At this point, 4 additional articles were excluded from further analysis because the content falls outside the scope of this research. Of these 4 articles excluded, 2 studies were eliminated because they covered quotation errors in the applied health sciences (nursing and manual therapy) and 1 study was excluded for examining quotation errors in promotional material. At this stage, 31 articles from the initial PubMed/MEDLINE search remained for analysis. In order to ensure comprehensive coverage, additional sources were identified by hand-searching the bibliography of each of the remaining articles and checking both similar and citing articles through PubMed/MEDLINE and Google Scholar.[10–12] The abstracts of relevant articles were examined and the complete text of relevant articles was collected. From these subsequent searches, an additional 19 articles were added to the 31 articles, for a total of 47 articles on misquotation in medical research journals. At this point, 3 literature reviews on quotation errors that did not have any original data were excluded from further analysis,[5, 7, 9] 2 sources were conference presentations[13, 14] and therefore were excluded because of the preliminary nature of conference data,[15, 16] 1 study was excluded for examining quotation errors in letters (commentaries),[17] which covered both a unique article type and was published as a letter that was not subject to comprehensive peer review, and 1 study was excluded for examining quotation errors in review (not primary research) articles.[18] At this stage, 40 original articles on quotation errors in the primary medical literature were subjected to qualitative analysis. Of these, 15 articles met the selection criteria (described below) for inclusion in the primary quantitative analysis. Another 5 articles were added to the supplemental analysis. The database and hand searches were repeated on April 1 and July 10, 2017, which did not result in any new original studies meeting the selection criteria.

### Selecting original studies and analyzing research design

Based on the above search strategy, 40 original studies of quotation errors in the primary medical literature (original research articles) were identified. These articles were grouped by the research design and generalizability of the findings as intended by the authors of these articles. Examination of these 40 articles revealed a total of 6 general categories, which were organized in increasingly specificity and therefore are decreasingly representative of the entire medical literature. As shown in Table 1, the categories consist of: (1) representative study of the entire medical literature, (2) representative study of a particular medical specialty, (3) non-representative study examining a single journal, (4) non-representative study examining 2 journals in a focused geographic region, (5) non-representative study examining an individual “fact” throughout the medical literature, and (6) anecdotal account or commentary (often of a particular article misquoting a previous source). Of these categories, only the representative investigations (categories 1 and 2) were included in the primary quantitative analysis because these were the only studies designed to represent the medical literature. Of these studies, only 1 investigation was designed to represent the broad medical literature (category 1). The majority of the studies (93%, 14/15) focused on error rates within a single medical specialty (category 2), which examined quotation errors in samples of specialty journals. Of the studies in category 2, only 1 study employed random selection of journals within the specialty and thus would be considered representative statistically (category 2a). Most original studies (categories 1 and 2b) were not truly representative of the field or specialty but were labeled “pseudo-representative” studies because the authors of each study deliberately selected journals to represent the field or medical specialty. Studies that included more specific selection criteria were excluded from the primary analysis. In a supplemental analysis, all studies of quotation errors with equivalent data (categories 1 to 4) were included in a broader recalculation of quotation error rate.

**Table 1:** Categories of studies of quotation (“factual”) errors in original research articles published in medicine. Categories are organized in order of increasing specificity and therefore are decreasingly representative of the entire medical literature.

Category	Stated Goal of Article and Intended Generalizability^a^	General Research Design	Strategy for Selection of Sample	No. of Articles^b^	Sources
**Representative Studies**					
1	Examining the entire medical literature	Quantitative	A “pseudo-representative” study, which had purposive (deliberate) selection of journals by the study authors (with the intent to represent the medical literature)	1	[6]
2	Examining a particular medical specialty	Quantitative	Category 2a: A “representative” study, which had true random selection of journals indexed within a medical specialty	1	[19]
			Category 2b: “Pseudo-representative” studies, which had purposive (deliberate) selection of journals by the study authors (with the intent to represent the journals within a medical specialty)	13	[20–32]
**Case Studies**					
3	Examining 2 journals in a focused geographic region	Quantitative	Purposive (deliberate) selection of 2 medical journals^c^ published within a defined geographic region^d^ for comparison to the larger corpus of medical literature (data not presented as representative of the broader medical literature)	2	[33, 34]
4	Examining a single journal	Quantitative	Purposive (deliberate) selection of a specific journal for comparison to the larger corpus of medical literature (data not presented as representative of the broader medical literature)	5 (2 identified as pilot studies[8, 35] & another 2 in Dutch^e^[36, 37])	[8, 35–38]
5	Examining a specific “fact” throughout the medical literature	Both quantitative and qualitative	Purposive (deliberate) selection of a particular “fact” that is widely reported in the medical literature	10	[39–48]
**Anecdotal Accounts and Commentaries**					
6	Observation or commentary (often in response to a particular article misquoting a previous source)	Qualitative	None, discovery occurs by chance observation	8	[1, 49–55]

^a^ As stated/presented by the authors of the original study.

^b^ Through July 10, 2017. Includes only published journal articles (in which the research studies were subject to the full peer review process) or, for anecdotal accounts, editorials/commentaries. Notably, this excludes preliminary data presented at conferences[13, 14] (which excludes data from one conference presentation[14] that was included in all previous reviews and data from another conference presentation[13] that was included in 2 (of 3) previous review[5, 8]).

^c^ “Geographic region” is defined as journals published within a specific country or a limited, defined geographical region. Notably, the authors of these studies defined the geographic region as part of the study design and compared the data to the broader medical literature.

^d^ By coincidence, the 2 regional studies both examined 2 medical journals. A notable distinction between a regional study (category 3) and a journal-specific analysis (category 4) is that the regional studies examined the accuracy of quotations in more than 1 journal.

^e^ Language barriers prevented analysis of articles not in English.[36, 37] These studies were categorized by information presented in the abstracts (which were available in English).

To my knowledge, this is the first systematic review that distinguishes studies of quotation error by research design. Although most original studies did not have truly random selection of journals in medicine (or a medical specialty), the authors of studies in categories 1 and 2b positioned the research articles as representative studies of the field (or specialty) and these are the most representative sources available for data on quotation accuracy throughout the field. The average of these studies, which is presented here, provides a more specific estimate of quotation errors in original research articles in medicine.

### Assigning consistent definitions and criteria for quotation errors

Since the original studies examined (from categories 1, 2a, and 2b) used different criteria for quotation errors, the next stage of this investigation was to apply consistent definitions for quotation errors in the original studies examined. First, I defined “content errors” as an informative or “factual” inaccuracies and distinguished these errors from “source errors,” which are secondary (or indirect) citations. Although source errors are procedurally improper, the quotations were not evaluated for informational or content accuracy and thus may not represent a “factual” inaccuracy. To focus on information accuracy (rather than the process of quotation by authors) and to ensure consistency of data between studies, I reevaluated the raw data reported in the original studies and separated source errors from content errors. The distinction between content and source errors was inconsistent in the original studies. Of 15 articles included in the quantitative analysis, 4 studies included secondary citations (source errors) as quotation errors, [22, 26, 31, 32,] 4 studies reported secondary citations separately,[6, 19, 27, 30] and 7 studies did not report secondary citations.[20, 21, 23–25, 28, 29] When the raw data of secondary citations was provided in the original study,[22, 26, 31] I recalculated the content errors to exclude these source errors. I was unable to separate source errors from the content errors in 1 study[32] because the original data were not provided at a sufficient level of detail. Excluding source errors from 3 of the 4 studies slightly lowered the total quotation error rate from the original articles and made the criteria of content errors comparable to other studies.

Second, I applied consistent criteria for subcategorizing content errors into major and minor errors. The criteria for major and minor errors is more standardized throughout the original studies, although some minor differences existed. (The most notable difference in the criteria is that source errors are considered minor errors in approximately half of the studies.) As summarized in Table 2, a major content error was a cited assertion in which the referenced source either failed to substantiate, was unrelated to, or contradicted the assertion.[6, 23, 24, 30] In contrast, a minor error was an oversimplification, a generalization, or a trivial inaccuracy that did not change the intended meaning of the original authors.[6, 23–25, 30] The percent of major and minor errors was calculated based on the total number of content errors.

**Table 2:** Types of content quotation errors.

Type of Error	Criteria	Example of Error		
		Cited Assertion	Analysis	Source of Example
**Major**	Cited reference failed to substantiate assertion	The most common shapes of apm (= anterior papillary muscle) were conical and flat-topped, according to Victor’s classification (15) used for papillary mm, of the left ventricle.	Victor and Nayak do not mention such a classification. Moreover, they dealt with the right ventricle.	[30]
	Cited reference was unrelated to assertion	The article by Lowe is cited to support an increased risk of esophageal cancer with alcohol consumption.	The article by Lowe is about treatment and contains absolutely no mention of etiology.	[24]
	Cited reference contradicted assertion	The average blood levels seen in our population (of human subjects) are below that usually associated with renal insufficiency.	The cited reference, a study of lead poisoning in rats, reported that in adult rats poisoned with lead when young, renal insufficiency persisted even after blood lead levels had fallen to normal.	[23]
**Minor**	Drawing conclusions that the authors of the cited reference were unwilling to do, an oversimplification, or a generalization (n.b., assertion does not significantly change the original assertion)	No association was found between the level of serum retinol and the subsequent development of cancer.	The cited reference found no association between serum retinol and *breast* cancer.	[23]
	Trivial inaccuracies in assertion or inaccuracies that do not change the meaning of the quoted source[6] (e.g., errors in patient numbers or some percentages)[25]	42 patients	Should be 42 abscesses in 40 patients	[6]

### Recalculating quotation error rates

For each study, the number of errors reported was divided by the quotation sample size, to yield a new estimate of the actual percent of quotation errors. The majority of original studies on quotation errors did not report the actual percent of quotations with errors to the total number of quotations examined. Rather, most original studies calculated the mixed metric of quotations with errors divided by the number of references selected for investigation from the work-cited list. This mixed metric was then multiplied by 100 and presented as a “percent.” However, there was not a one-to-one correspondence between quotations in the articles and references in the work-cited list, which means the mixed metric was not a true percent. Furthermore, the use of the mixed metric was not universal throughout the original studies. In 4 studies,[22, 26, 30, 31] the authors reported both the prevalent measure of quotation errors to references selected as well as the true percent of quotation errors to quotations examined. In 1 study,[6] the authors only reported the percent of quotations with errors to the total number of quotations selected for analysis.

To recalculate the quotation error rate, I collected the raw data from the original studies for the number of quotations examined and the number of quotation errors that fit the descriptions in Table 2. For studies that did not report the number of quotations examined, which was a large number of studies, I calculated the ratio of quotations to references selected. This ratio was used to estimate the total number of quotations examined in those studies. The percent of quotation errors was calculated by dividing the number of quotation errors by the number of quotations examined.

In 10 studies included in the primary analysis (and 4 studies in the supplemental analysis), the percent of quotation errors is based on an estimate of the number of quotations examined because these studies did not report the total number of quotations selected (the only sample size information reported in these studies was the number of references selected). For these studies, I estimated the number of quotations examined by using the average ratio of quotations to references from 4 studies[22, 26, 30, 31] that reported these data (Table 3). Based on these 4 studies, the mean ratio of quotation to references was 1.66 (1470 quotations/888 references). The percent of quotation errors was then recalculated for the other studies by multiplying the number of references selected by 1.66 to estimate the quotation sample size. The percent error introduced by estimating the quotation sample size was determined by comparing the quotation error rates using the estimated sample size of quotations to the actual quotation error rates in the 4 studies[22, 26, 30, 31] where the data were available. The rate of source errors was recalculated using the same procedure as described for content errors.

**Table 3:** Ratio of quotations examined to references selected.

Specialty	Year of Sample	Quotations Examined (n)	References Selected (n)	Ratio of Quotations to References	Source
Anatomy	2001	272	199	1.37	[30]
Dermatology	1992	392	240	1.63	[26]
Orthopedic	2007–8	398	200	1.99	[22]
	2009	408	249	1.64	[31]
Total		1470	888	1.66	

## Results

Of 43 original articles on quotation errors in the medical literature published through July 2017, 15 met the selection criteria for the primary analysis as quantitative investigations of quotation errors in original research articles that were representative of journals covering general medicine or a particular specialty. In these 15 articles, a total of 3,337 references and 5,535 quotations (estimated) were individually evaluated for accuracy and appropriateness by subject-matter experts. After applying consistent definitions throughout the original studies, the initial ratio of quotation errors to references selected was 0.241 (0.163 to 0.319) at a 95% confidence interval (Table 4). The recalculation of total rate of quotation errors to quotations analyzed is 14.5% (10.5% to 18.6% at a 95% confidence interval). The percent error introduced by estimating the quotation sample size is 3.4% (S1 Table). Of the content errors, 64.8% (56.1% to 73.5% at a 95% confidence interval) are major errors and 35.2% (26.5% to 43.9% at a 95% confidence interval) are minor errors. In the 7 studies reporting quotation source errors (see “source errors” in Table 4), the error rate of improper secondary (or indirect) citations (rather than citation to the primary research article) was 10.4% (3.4% to 17.5% at a 95% confidence interval). In the supplemental analysis of studies in categories 1 to 4 (S2 Table), the quotation error rate is estimated to be 0.236 (0.156 to 0.315 at a 95% confidence interval) and 14.2% (9.8% to 18.6% at a 95% confidence interval).

**Table 4:** Prevalence of content and source errors in original research articles in medicine by journal specialty.

Journal Specialty	Publication Year of Sample	Content Errors				Source Errors	Source
		Ratio of Quotation Errors to References Selected	Quotation Errors in Primary Research Articles (%)	Major Errors (%)	Minor Errors (%)	Improper Secondary Citations in Primary Research Articles^a^ (%)	
Anatomy (Gross)	2001	0.261 (52/199)	19.1 (52/272)	94.2 (49/52)	5.8 (3/52)	23.9 (65/272)	[30]
Burn	2006	0.142 (16/113^b^)	8.5^c^ (16/188)	50.0 (8/16)	50.0 (8/16)	-	[20]
Dermatology	1992	0.292 (70/240)	17.9 (70/392)	51.5 (34/70)	48.5 (36/70)	3.3 (13/392)	[26]
Emergency Medicine	1991	0.352 (51/145)	21.2^c^ (51/241)	82.4 (42/51)	17.6 (9/51)	24.9^c^ (60/240.7)	[27]
General Medicine	1984	0.239^d^ (36/151)	14.4^e^ (36/250)	44.4 (16/36)	55.6 (20/36)	6.0^f^ (15/250)	[6]
Ophthalmology	2003	0.250 (50/200)	15.1^c^ (50/332)	60.0 (30/50)	40.0 (20/50)	-	[21]
Orthopedic	2007–8	0.680 (136/200)	34.2 (136/398)	80.9 (110/136)	19.1 (26/136)	7.5 (30/398)	[22]
	2009	0.293 (73/249)	17.9 (73/408)	49.3 (36/73)	50.7 (37/73)	2.0 (8/408)	[31]
Otolaryngology/Head and Neck Surgery	1997	0.170 (26/153)	10.2^c^ (26/254)	65.4 (17/26)	34.6 (9/26)	-	[25]
Psychiatry	1997	0.068 (10/147)	4.1^c^ (10/244)	80.0 (8/10)	20.0 (2/10)	-	[29]
Public Health	1986	0.300 (45/150)	18.1^c^ (45/249)	51.1 (23/45)	48.9 (22/45)	-	[23]
Radiology	1993	0.095 (9/95)	5.7^c^ (9/158)	77.8 (7/9)	22.2 (2/9)	-	[28]
Surgery	1987	0.292 (40/137)	17.6^c^ (40/227)	92.5 (37/40)	7.5 (3/40)	-	[24]
	2004	0.078^g^ (20/258)	4.7^c^^,^^g^ (20/428)	80.0 (16/20)	20.0^§^ (4/20)	-^a^	[32]
	2007	0.189 (170/900)	11.4^c^ (170/1,494)	51.8 (88/170)	48.2 (82/170)	11.4^c^ (170/1,494)	[19]
**Total Rate**^h^ [95% confidence interval]		**0.241** (804/3,337) [0.163 to 0.319]	**14.5%** (804/5,535) [10.5% to 18.6%]	**64.8%** (521/804) [56.1% to 73.5%]	**35.2%** (283/804) [26.5% to 43.9%]	**10.4%** (361/3,455) [3.4% to 17.5%]	

^a^ Values are only provided for studies investigating and reporting improper secondary (indirect) quotations. Studies that do not have values did not evaluate secondary citations (except for one study[32] that included source errors in the measure of content errors, but did not provide sufficient resolution of the data to distinguish source errors from content errors).

^b^ The original article[20] provided conflicting information regarding the total number of references. A total number of 117 references was reported investigated although the authors noted that 4 of the original source articles could not be retrieved. Since the original text of an article is required for quotation analysis, a prior review article[5] used 113 as the number of references analyzed, which was also used in this analysis. The corresponding author of the original study did not respond to a request for clarification.

^c^ Errors per quotation examined were estimated by multiplying the number of references selected by 1.66, which was the ratio of quotations to references (1,470/888 = 1.66) that was calculated from 4 studies[22, 26, 30, 31] that reported both data (see Table 3).

^d^ The original study[6] was unique in that quotations were selected randomly from text rather than references selected from the work-cited list. Therefore, the authors did not report the ratio of quotation errors to references selected. The number of references was estimated based on the ratio of 1.66 quotations to references.

^e^ For this analysis, the data from the journal selected for high proportion of review articles, *British Journal of Hospital Medicine*,[6] was excluded to maintain consistency in article types examined.

^f^ Ratio per quotation (not per reference in the work-cited list).

^g^ Includes secondary citations as minor content errors (the number of secondary citations was unable to be distinguished from the minor content errors).

^h^ Calculated by dividing the total number of errors by the total sample size.

## Discussion

In this analysis, I examined the methods of studies of quotation errors in the medical literature and recalculated the quotation error rates from the original data after applying a consistent definition of errors across studies and basing the percent on quotations examined. The main outcome of this investigation is a more precise estimation of the accuracy or information integrity of cited assertions (i.e., the cited “facts”) in original research articles published in medical journals. In the primary analysis, I estimate that 14.5% (10.5% to 18.6% at a 95% confidence interval) of cited assertions in original research articles in medical journals are inaccurate. This estimate has a more specific definition of the “medical literature,” as original research articles, and is approximately 5–10% lower than estimates reported in previous reviews.[5, 7, 8] Notably, this rate is also approximately 10% lower than the mixed-metric ratio of quotation errors to references, which was 0.241 (0.163 to 0.319 at a 95% confidence interval) and was often multiplied by 100 and incorrectly reported as a percent in previous studies. Thus, the lower rate of quotation errors estimated here is predominantly due to adjusting the metric from the mixed-metric ratio to a true percent of quotations. This qualitative study also had several other differences in the approach in comparison to previous reviews (Table 5). The differences include the selection of studies used for the analysis, which provides a more specific rate of quotation errors based on a more restricted definition of the medical literature, as well as reanalysis of the original data to adjust for consistency within the original studies.

**Table 5:** Comparison of reviews on quotation errors in the medical literature.

	Total Quotation Error Rate Reported (%^a^)	Study Selection						Data Analysis		
		Year Literature Search Conducted	No. Studies Included in Analysis	Excludes Non-Representative Investigations^b^	Data from Primary Research Articles Only (Subject to comprehensive peer review)	Scope of Scientific Literature Examined	Article Types Examined for Quotation Errors	Applies Consistent Definitions of Quotation Errors between Studies	Adjusts Original Data for Equivalency between Studies	Recalculates “Percent” as Quotations with Errors to Quotations Examined
**This Review**	Primary Analysis: 14.5 (Content Errors) 10.4 (Source Errors)	2017	15	Yes	Yes	Medical (includes Public Health[23])	Original research articles^c^	Yes	Yes^d^	Yes
	Supplemental Analysis: 14.2 (Content Errors)	2017	20	No	Yes					
**Jergas & Baethge**[5]	25.4^a^	2014	28	No	No, includes data from a conference presentation[14] and letter[17]	Medical and Applied Health (includes Manual Therapy,[56] Nursing,[57] and Public Health[23])	All reported types^e^	No	Limited^f^	No
**Mertens & Baethge**[8]	19.7^a^	2011	26	No	No, includes data from a conference presentation[14] and letter[17]	Medical and Applied Health (includes Manual Therapy,[56] Nursing,[57] and Public Health[23])	All reported types^e^	No	No	No
**Wager & Middleton**[7]	20^a^	2006–7	20	No	No, includes data from conference presentations[13, 14] and a letter[17])	Medical and Applied Health (includes Manual Therapy,[56] Nursing,[57] and Public Health[23])	All reported types^e^	No	No	No
**Riesenberg & Dontineni**[9]	0–58^g^	2000	12	No	No, includes data from a letter[17]	Medical and Applied Health, (including Nursing[57] and Public Health[23]), and Veterinary[58]	All reported types^e^	Yes	NA^h^	NA^h^

^a^ “Percent” reported in previous review articles is based on inconsistent definitions and calculations of quotation errors in the original studies. (For more detail, refer to the Methods section.)

^b^ As intended by the authors of the original study. Prior reviews on quotation errors combined estimates of the overall rate of quotation errors from studies with different research designs and intended generalizability.

^c^ Excludes data from other article types when the raw data was available. A minor subset of other article types (such as review articles) were included in this analysis because some original studies included these articles in their analysis.

^d^ Qualitative evaluation of methods followed by recalculation of quotation error rates from original data (see Methods section).

^e^ Previous reviews of quotation errors did not limit analysis to a specific type of article in the medical literature. Therefore, previous reviews included studies examining quotation errors in original research articles (primarily, as in this review), but the reported rate of quotation errors in previous reviews also includes data from studies of quotation accuracy in letters[17] and review articles.[18]

^f^ Statistical measure of bias, which was not included in primary report of quotation errors. Risk of bias was determined by two variables: (1) random selection of references used for evaluation and (2) a minimum of 2 researchers that independently rated the quotation accuracy.[5]

^g^ Only a range was provided (no measure of central tendency). The methods in the review article were not sufficiently described to determine how the “percent” was calculated.

^h^ Information not available.

Unlike previous reviews, the rate of quotation errors presented here only includes data from published, peer-reviewed studies of quotation errors in original research articles in medical journals (excluding preliminary data from conferences and letters, and data from other fields). Additionally, the primary analysis only included studies that were presented by the original authors as representative studies of quotation errors in the medical field of specialties (Table 1), which excluded case studies that were designed to investigate specific instances of quotation errors. The exclusion of categories 3 and 4, which were limited investigations of either 2 regional medical journals or a single journal, from the primary analysis is debatable. The primary reason for excluding studies in categories 3 and 4 is because this examination is a qualitative review of research methods and, as such, adding the studies in category 3 and 4 would result in comparison of studies with dissimilar designs. Yet, a quantitative argument for including all similar data is valid. Therefore, the data from the 5 studies in categories 3 and 4 were included in a supplemental analysis (S2 Table). Including the data from these studies did not change the overall quotation error rate, which was estimated to be 0.236 (0.156 to 0.315 at a 95% confidence interval) quotations with errors to references selected and 14.2% (9.8% to 18.6% at a 95% confidence interval) quotations with errors.

The main difference between this review and previous reviews is that the calculation of quotation errors here is the “percent” of quotation errors of quotations examined rather than a mixed-metric ratio of quotation errors to references selected. Calculating the actual percent of quotation errors required reanalysis of the original data, rather than using the inconsistently calculated error rates in the original studies. In most original studies and prior reviews, the “percent” of quotation errors was a misleading label for the ratio of two different measures: the number of in-text quotations with errors divided by the number of references selected for analysis from the work-cited list. The resulting ratio was then multiplied by 100, which might be the origin of the “percent” label. The problem with the ratio of quotation errors to references, when presented as a “percent,” is that a one-to-one correspondence between quotations and references does not exist in medical articles, even though the two are not entirely independent. Often, a reference listed in the work-cited list may be cited multiple times within an article and also might correspond to several different assertions (e.g., one study[26] mentioned an extreme case in which a single reference was associated with 13 in-text quotations). Alternatively, a reference may be inadvertently included in the work-cited list but not quoted in the text (as mentioned in the same study[26]). In many studies (and carried through to other reviews), the mixed-metric ratio reported (inaccurately called a “percent”) inflated prior estimates of quotation errors because the number of references, the denominator, was less than the actual number of quotations examined. In contrast, this review provides an overall measure of quotation errors that is more accurately called a “percent.”

Further commentary is necessary in regards to combining the single study[6] that was designed to represent the entire medical literature (a category 1 study) with the 14 studies that were designed to represent individual medical specialties (category 2 studies). The primary reason for including the de Lacey et al. study[6] representing the broad medical field was because that was only study that examined quotation errors in the major general medical journals (*BMJ*, the *Lancet*, the *NEJM*) and the study might be viewed as a focused study of quotation error rates in general medical journals. Therefore, the primary analysis may be viewed as an review of studies with different medical journal focuses, which means that the journal specialities listed in Table 4 are roughly equivalent. A secondary reason was to partially weight the overall mean of quotation errors towards the rate reported in the only study designed to estimate the rate of quotation errors throughout the medical field. Notably, de Lacey et al.[6] reported a quotation error rate of 14.4% (in original research articles), which is very close to the rate 14.5% calculated in the primary analysis here. One of the primary limitations to this analysis was that the percent of quotation errors was recalculated from the available raw data. The total number of quotations had to be estimated in studies that did not provide the exact number of quotations examined. This estimate was based on the assumption that the average ratio of quotations to references was similar in all studies of quotation errors in the medical literature. Thus, the ratio of quotations to references was calculated from 5 studies that reported both the number of quotations and references. This ratio was then used to estimate the quotation sample size in studies that only reported the number of references for the sample size. Although the estimate of quotations examined introduced a degree of uncertainty, the percent error introduced is 3.4%, which is within the 3.9% margin of variability for the percent of quotation errors (at a 95% confidence interval).

Another limitation in the calculation of quotation errors was that the orginal studies had an inconsistent, often biased, selection of a single quotation from multiple quotations associated with a single reference. In most original studies, the authors selected references from the work-cited list (rather than quotations within the article) to examine. If multiple quotations were associated with a single reference, different studies had different methods to select only one quotation to include in the calculation of quotation errors. Of the 10 studies (in the primary analysis) that reported the ratio of quotations with errors to references (rather than the actual percent), 4 studies[20, 23, 25, 32] reported the most substantial error (a major error if both major and minor existed), 1 study[27] reported the lowest ranking error, 1 study[21] reported the results from first instance of a quotation, and 4 studies[20, 24, 28, 29] did not describe the selection process for selecting a single reference from multiple in-text quotations. Because the authors of the original studies did not provide sufficient resolution of the data, I was unable to adjust for the biased selection of a single quotation from multiple quotations associated with the same reference. Thus, the quotation error rate estimated in this review may be slightly higher (and more extreme leaning towards major errors) than the actual quotation error rate.

A broader issue in this analysis, as well as all research on quotation errors, is that experts must evaluate and categorize each quotation in the context of the referencing article and compare the assertion to information provided in the original source. Having experts evaluate information integrity of quotation errors in the medical literature confers both strengths and limitations to the design of the original studies. The primary strength of the design, as well as a practical explanation for the trend of field-specific studies, is that the researchers of each study were experts within the specialty and therefore are able to evaluate and compare the nuances of assertions to the data and statements in the original research article. Generally, each assertion was evaluated by multiple experts and the classification of quotation error was determined by consensus.[19, 20, 22, 27, 31, 32] Among the studies that provided the frequency of inter-rater agreement, a good to excellent agreement between experts was reported at 83.3%–87.5%,[27] 87.9%,[22] and 90.4%.[31] Yet, several studies also noted some degree of difficulty,[20, 27, 31] subjectivity,[31, 32] and disagreement[19, 22, 27, 31] among the authors while evaluating assertions. In contrast to the strength of good to excellent inter-rater agreement for categorization of quotation errors, a resulting limitation was that each original study examined a single specialty corresponding to the expertise of the authors. Thus, the quotation error rate may not generalize to other specialties beyond the ones examined. As mentioned by Jergas and Baethge[5] in a prior review, the original studies cover a broad range of medical specialties but are incomplete and may not be representative of the entire medical field. However, Jergas and Baethge[5] also predict that including a few additional original research studies in other specialties would not necessarily make a meaningful change to the overall quotation rate due to the wide range of data from individual specialties (i.e., 4.1% to 34.2% in the recalculations here). The primary analysis reported here does have a smaller sample size (n = 15) than the Jergas and Baethge[5] review (n = 28 studies in the main analysis), so the overall rate reported here would be influenced more by additional studies. Yet, adding 5 studies to the 15 studies only resulted in a minor difference between the quotation error rate of 14.5% in the primary analysis and 14.2% in the supplemental analysis. But, being limited by the scope of the original studies and availability of data, I believe that this review remains a more accurate estimate of the percent of quotation errors in original research articles in medicine.

Despite the limitations of comparing error rates between individual specialties, comparison between different specialties is prevalent in the original studies. However when the data between similar studies of quotation errors is compared in the same specialties (e.g., orthopedic[22, 31] and surgery[19, 24, 32] fields), there is large difference in the quotation error rates. Thus, the validity of comparison between fields may be vastly inaccurate. For example, in 2 studies of the orthopedic literature with very similar sampling procedures and journals (including some of the same journals), an approximately two-fold difference in the quotation error rates were reported. In a 2007–8 sample of 200 references from 4 orthopedic journals,[22] one study reported a quotation error rate of 38% (30.1% to 47.0% at a 95% confidence interval). (The content quotation error rate for this study[22] data was recalculated here at 34.2%.) In a 2009 sample of 249 references 5 orthopedic journals (2 journals, 40%, were the same as the previous study[22]), another study[31] reported a quotation error rate of 20% (16% to 24% at a 95% confidence interval). (The content quotation error rate for this study[31] data was recalculated here at 17.9%.) Yet, quotation errors within the same journals were consistent. A sample of quotations from the *American Journal of Bone and Joint Surgery* in 2007–8 had a quotation error rate of 22.8% compared to a 2009 sample with a quotation error rate of 20.0%. A sample of quotations from the *British Journal of Bone and Joint Surgery* in 2007–8 had a quotation error rate of 15.8% compared to a 2009 sample with a quotation error rate of 16.2%. Since these studies were published in 2010 and 2013, publication of the first study was not likely to have influenced the observed rate in the 2009 sample of the latter study. The point is that the two-fold difference of quotation error rates in similar specialties might arise from the selection of different journals within the specialties. However, despite the concern that the comparison between studies and specialties may not be appropriate, I believe that the quotation error rate of the entire medical field reported here remains a reasonable estimate of quotation errors because the variability arising from sampling error in each original study would be, theoretically, adjusted through averaging. Still, the percent quotation error rate reported here is the most reliable estimate possible based on the current data available.

In regards to the identifying and accessing original studies on quotation errors in medicine, I am reasonably confident that the corpus of studies included in this analysis was exhaustive or nearly exhaustive. For this analysis I conducted an extensive literature search in PubMed/MEDLINE to identify an initial set of articles, hand-checked the reference lists of all articles, and concluded by checking for related articles in both PubMed/MEDLINE and Google Scholar databases. This analysis included the same studies in the quantitate analysis as the other reviews,[5, 7, 8] which used slightly different search procedures. Again, Jergas and Baethge[5] predicted that a few studies, which may have been missed, are unlikely to make a meaningful change to the overall quotation rate. Again, adding 5 category 3 and 4 studies in the supplemental analysis to the primary analysis of 15 studies resulted in minimal change to the average rate of quotation errors.

Despite estimating a lower rate of quotation errors than prior analyses,[5, 7, 8] I believe that the rate of content quotation errors is still alarmingly high in the medical literature. The estimated rate reported here, 14.5%, which spans nearly 25 years of data, is notably similar to the 14.4% quotation error rate reported[6] in a sample from 1984 (after excluding the data from the journal selected to include a high number of review articles). These rates suggest that reporting quotation errors in the medical literature has not influenced authors or changed publication practice throughout the field. Indeed, Jergas and Baethge[5] reached the same conclusion. Yet, some of the lowest rates of quotation errors (at 4.1%,[29] 4.7%,[32] and 5.7%[28]) might indicate that a much lower overall baseline error rate is feasible. Possibly these samples have some unique characteristics in the communication and publishing process that results in a lower overall rate. Indeed, the consistency of error rates of the 2 sets of journals in the orthopedic literature suggests that quotation error rate might be journal specific.

Furthermore, the distribution of content errors (64.8% major to 35.2% minor errors) is particularly concerning. The disproportionate amount of major errors might suggest that authors are not reading[3, 4, 23, 24, 26, 50] or possibly comprehending[5, 41] prior research. In other instances, major errors may even suggest a deliberate attempt by authors to mislead readers,[1, 59, 60] which includes peer reviewers and editors, because major errors are significantly different than the information provided in the original studies. In some cases, inclusion of inappropriate citations might be due to citation manipulation in which the authors intend to increase citation counts of unrelated articles.[26, 28] In contrast, minor errors (the trivial inaccuracies), which are one-third of content errors, might be more a result of carelessness. For physicians and other readers, this relatively high rate of gross inaccuracy in the cited “facts” means that readers must be somewhat skeptical of cited assertions in original medical research articles and they should access the original source to verify the information before taking or revising practice based on a secondary report. Furthermore, false beliefs introduced through content quotation errors also can negatively influence future research and public policy, particularly if coupled with improper secondary citation practices that perpetuate such errors.

In the studies reporting source errors, the rate of improper secondary (indirect) citations was 10.4%, which was similarly adjusted as a percent rather than the weighted metric of improper secondary citations to references selected in the work-cited list. Although secondary citation is procedurally improper, this measure does not provide any insight into the integrity of the information cited since the authors of the original studies did not trace the statements further and analyze for accuracy. Thus, source errors are subordinate to content errors and should be reported separately from content errors in future studies.

## Supporting information

S1 Table Comparison of estimated versus actual errors per quotations. Data excludes references to secondary sources (indirect citations).
(DOCX)

S2 Table Supplemental analysis of quotation errors from studies categorized as 1, 2, 3, and 4. (DOCX)

S1 File PRISMA checklist. This review includes the recommended content provided in the PRISMA checklist.
(DOC)
