# Supplementary material for: Accuracy of cited “facts” in medical research articles: A review of study methodology and recalculation of quotation error rate
Source: PLoS One. 2017 Sep 14;12(9):e0184727. doi: 10.1371/journal.pone.0184727 (PMC5599002; doi:10.1371/journal.pone.0184727)
Supplement: S1 Table — Data excludes references to secondary sources (indirect citations). (DOCX) [file pone.0184727.s001.docx]

S1 Table. Comparison of Estimated Versus Actual Errors Per Quotations. Data excludes references to secondary sources (indirect citations).

| **Specialty** | **Year of Sample** | **Estimated Error Rate**  **(%)** | **Actual Error Rate**  **(%)** | **Percent Error**  **(%)** | **Source** |
| --- | --- | --- | --- | --- | --- |
| Anatomy | 2001 | 15.8 | 19.1 | -21.0 | [30] |
| Dermatology | 1992 | 17.6 | 17.9 | -1.6 | [26] |
| Orthopedic | 2007–8 | 41.1 | 34.2 | 16.7 | [22] |
|  | 2009 | 17.7 | 17.9 | -1.1 | [31] |
| Total | | 23.0 | 22.3 | 3.4 |  |
