# Supplementary material for: Accuracy of cited “facts” in medical research articles: A review of study methodology and recalculation of quotation error rate
Source: PLoS One. 2017 Sep 14;12(9):e0184727. doi: 10.1371/journal.pone.0184727 (PMC5599002; doi:10.1371/journal.pone.0184727)
Supplement: S2 Table — (DOCX) [file pone.0184727.s002.docx]

S2 Table. Supplemental Analysis of Quotation Errors from Studies Categorized as 1, 2, 3, and 4.

| **Study Group** | **Publication**  **year of**  **sample** | **Content errors** | | **Source** |
| --- | --- | --- | --- | --- |
|  |  | **Ratio of quotation errors to references selected** | **Quotation errors in primary research articles**  **(%)** |  |
| Category 1 & 2  (Primary Analysis) | 1984–2009 | 0.241  (804/3,337) | 14.7  (804/5,535) | Table 4 |
| Category 3 | 1990 | 0.225  (27/120) | 13.6%^a^  (27/199) | [34] |
|  | 1993 | 0.205  (41/200) | 12.3%^a^  (41/332) | [33] |
| Category 4 | 2002 | 0.085  (15/176) | 5.1%^a^  (15/292) | [38] |
|  | 2008 | 0.391  (18/46) | 23.6%^a^  (18/76) | [35] |
|  | 2010 | 0.531^b^  (16/30) | 32.0%  (16/50) | [8] |
| Total Rate^c^  [95% confidence interval] | | 0.236  (921/3,909)  [0.156 to 0.315] | 14.2%  (921/6,485)  [9.8% to 18.6%)] |  |

^a^ Errors per statement are estimated by recalculating the number of quotations examined from the number of references reported based on the ratio of quotations to references (1,470/888=1.66), which was calculated from total counts in 4 studies[22, 26, 30, 31] that reported the data (see Table 3).

^b^ Errors per reference are estimated by recalculating the number of references examined from the number of quotations reported based on the ratio of quotations to references (1,470/888=1.66), as above.

^c^ Calculated by dividing the total number of errors by the total sample size.
